# Reduction in soil CO_2_ efflux through alteration of hydrothermal factor in milk vetch (*Astragalus sinicus* L.)-rapeseed (*Brassica napus* L.) intercropping system

**DOI:** 10.3389/fpls.2022.1093507

**Published:** 2023-01-10

**Authors:** Quan Zhou, Anna Gunina, Jiao Chen, Yi Xing, Ying Xiong, Zhiming Guo, Longchang Wang

**Affiliations:** ^1^ College of Agronomy and Biotechnology, Southwest University/Key Laboratory of Eco-environments in Three Gorges Reservoir Region, Ministry of Education/Engineering Research Center of South Upland Agriculture, Ministry of Education, Chongqing, China; ^2^ Jiangxi Agricultural University/Key Laboratory of Crop Physiology, Ecology and Genetic Breeding, Ministry of Education, Nanchang, China; ^3^ Department of Environmental Chemistry, University of Kassel, Witzenhausen, Germany; ^4^ College of Agriculture, Henan University of Science and Technology, Luoyang, China; ^5^ Key Laboratory of Vegetation Restoration and Management of Degraded Ecosystems, South China Botanical Garden, Chinese Academy of Sciences, Guangzhou, China

**Keywords:** legume-brassica intercrops, greenhouse gas emission, soil temperature, soil moisture, SOM balance

## Abstract

**Introduction:**

Intercropping has a potential to reduce the CO_2_ emission from farmlands. Limited information is available on the underlying reasons.

**Methods:**

This study investigated the effect of milk vetch (*Astragalus sinicus* L.) (MV), rapeseed (*Brassica napus* L.) monoculture (RS) and intercropping (Intercrop) on soil CO_2_ emissions, moisture and temperature in a bucket experiment during 210 days from October 2015 to May 2016 on Chongqing, China.

**Results:**

The results showed that soil CO_2_ efflux of MV, RS and Intercrop was 1.44, 1.55 and 2.08 μmol·m^-2^·s^-1^ during seedling and stem elongation stages and 3.08, 1.59 and 1.95 μmol·m^-2^·s^-1^ during flowering and podding stages. At seeding and stem elongation stages Intercrop had 1.4 times higher soil CO_2_ efflux than the mean of MV and RS. In contrast, MVhad 1.6 times higher soil CO_2_ efflux than Intercrop thereafter, which shows it was inhibited if milk vetch presents as Intercrop only. Decreased sensitivity of soil respiration to temperature in 1.4 times and lower soil moisture by Intercrop were found compared to MV. Intercrop decreased soil moisture, especially at the seedling and stem elongation stages, compared to the monoculture. The fluctuation on soil respiration in RS and Intercrop was slight with changes in soil moisture.

**Conclusion:**

Thus, milk vetch-rapeseed system has a potential to decrease CO_2_ emission from farmland, however soil moisture should be regulated properly.

## 1 Introduction

The CO_2_ production from agriculture accounts for 23% of anthropogenic greenhouse gas emissions (Smith et al., 2007). If intercropping with legumes or cereals is introduced (Hu et al., 2016; Cui et al., 2019), this emission can be substantially reduced (Gan et al., 2011), because such systems have a high potential to sequester soil organic carbon (SOC) by reducing soil respiration (Chai et al., 2014; Hu et al., 2015). Namely, pea - maize or wheat - maize intercropping reduces soil respiration from maize strips during the growing season (Li et al., 2001; Qin et al., 2013); pea - oat intercropping reduces CO_2_ emission during the period with higher precipitation; barley - pea intercropping also results in 10% higher soil C sequestration than barley monoculture (Chapagain and Riseman, 2014).

Agricultural ecosystems increase SOC sequestration up to 4% if intercropping with legumes or cereals is introduced compared to crop monoculture (Cong et al., 2015). This is associated with: i) regulation of crop growth by intercropping, and thus, reduction of root exudates and following CO_2_ efflux (Dyer et al., 2012; Qin et al., 2013); ii) changes in the composition of microbial community structure and decrease in biomass and functional diversity under one of the species (Zhou et al., 2019b) or stimulation of soil microbial biomass growth under intercropping (Latati et al., 2017), and iii) the regulation of soil CO_2_ efflux by plant species composition, which may be suppressed by reduce of net primary production because of water availability shortage (Zhou et al., 2019a). Therefore, various intercropping systems (e.g., rapeseed (pea) - maize, wheat-soybean (maize)) can have the potential to reduce the soil CO_2_ efflux from farmland (Li et al., 2001; Chai et al., 2014).

Soil hydrothermal factors play an important role in CO_2_ efflux from farmland (Hursh et al., 2017). Temperature is the most important factor affecting soil respiration, and there is a positive relationship between them (Hursh et al., 2017). Plant community composition affects soil respiration's temperature sensitivity. The temperature sensitivity of soil respiration is affected by plant community composition (Mauritz and Lipson, 2021). Higher temperatures are generally expected to enhance soil C losses due to increased soil decomposition (Crowther et al., 2016; Bond-Lamberty et al., 2018). The sensitivity of respiration to temperature changes with the soil water content, substrate availability, and species composition (Geist and Lambin, 2004). The combined factors of soil temperature and moisture would better predict soil respiration (Feng et al., 2018). Soil moisture also strongly affects the changes in SOM (Jassal et al., 2008; Wang et al., 2014), respiration (Bouma et al., 1997), and microbial activity (Hallett and Young, 1999; Drenovsky et al., 2004), and the synergistic relationship between soil respiration and moisture can greatly increase or decrease the decomposition rate of SOM, depending on the direction of moisture change (Sierra et al., 2015). However, it is not yet clear how hydrothermal factors will be changed in the intercropping of the legume with brassica and what the response of CO_2_ emission will be.

Milk vetch (*Astragalus sinicus* L.) intercropping with rapeseed (*Brassica napus* L.) can enhance farmland productivity (Zhou et al., 2018), change the microbial community structure and decrease microbial biomass and functional activity in the rapeseed rhizosphere (Zhou et al., 2019b). To verify the potential of intercropping to reduce the CO_2_ emission, the soil respiration from milk vetch - rapeseed system was monitored together with the temperature and moisture for the entire development of crops and compared to the monoculture. Considering that intercropping can improve water use efficiency (Ren et al., 2017), and can decrease soil temperature (Gong et al., 2019) because of the large soil surface cover, it was hypothesized that soil CO_2_ efflux would be lower compared to the monocultures. The objective of the experiment was to explore the effects of intercropping on soil respiration and to determine the relationship between soil respiration and hydrothermal factors.

## 2 Materials and methods

### 2.1 Experimental site

**Figure 1:**
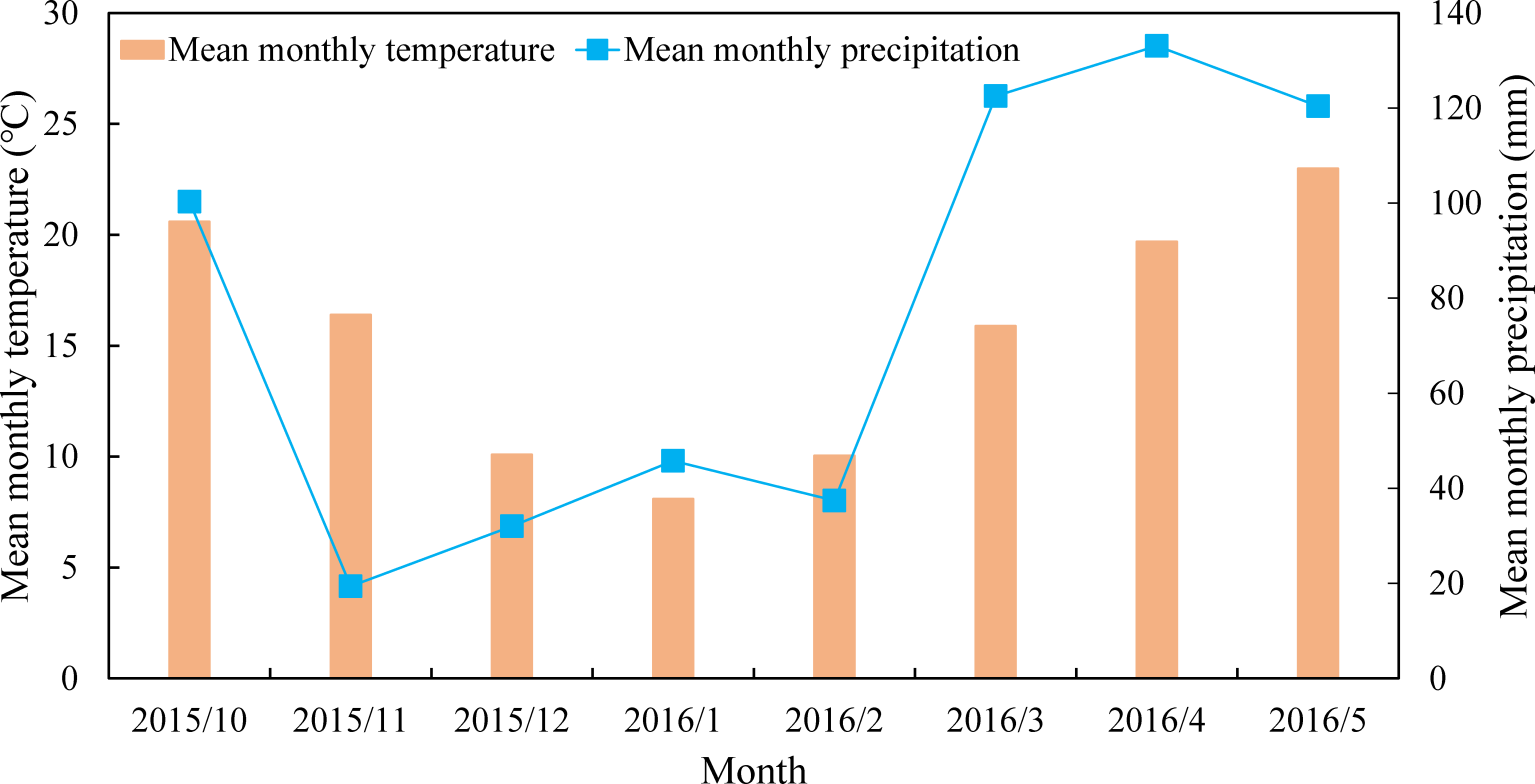
Mean monthly temperature and precipitation during the experiment.

The study was conducted from October 2015 to May 2016 on Southwest University experimental farm (29°81′N, 106°41′E), Beibei, Chongqing, China, which belonged to humid subtropical monsoon climate zone (**Figure 1**). Soils (0-15 cm) were classified as dystric Regosols with a pH of 6.30, the total C content of 8.6 g kg^-1^ and total nitrogen content of 0.97 g kg^-1^.

### 2.2 Experimental design

The experiment was conducted in buckets (0.7 m in height, 0.4 m diameter at the bottom, and 0.57 m at the top. V = 0.12 m^3^), that were installed outside. Soil for the experiment was collected at 0-15 cm depth from the Southwest University experimental farm. All soil was well-mixed after being air-dried. Each pot contained 50 kg of dry soil in which the fertilizers (0.10 g N kg^-1^, 0.10 g P_2_O_5_ kg^-1^ and 0.10 g K_2_O kg^-1^ dry soil) was mixed before sowing. Make sure all soil was compacted into the buckets so that the density was equal to reduce the effect on soil respiration.

**Figure 2:**
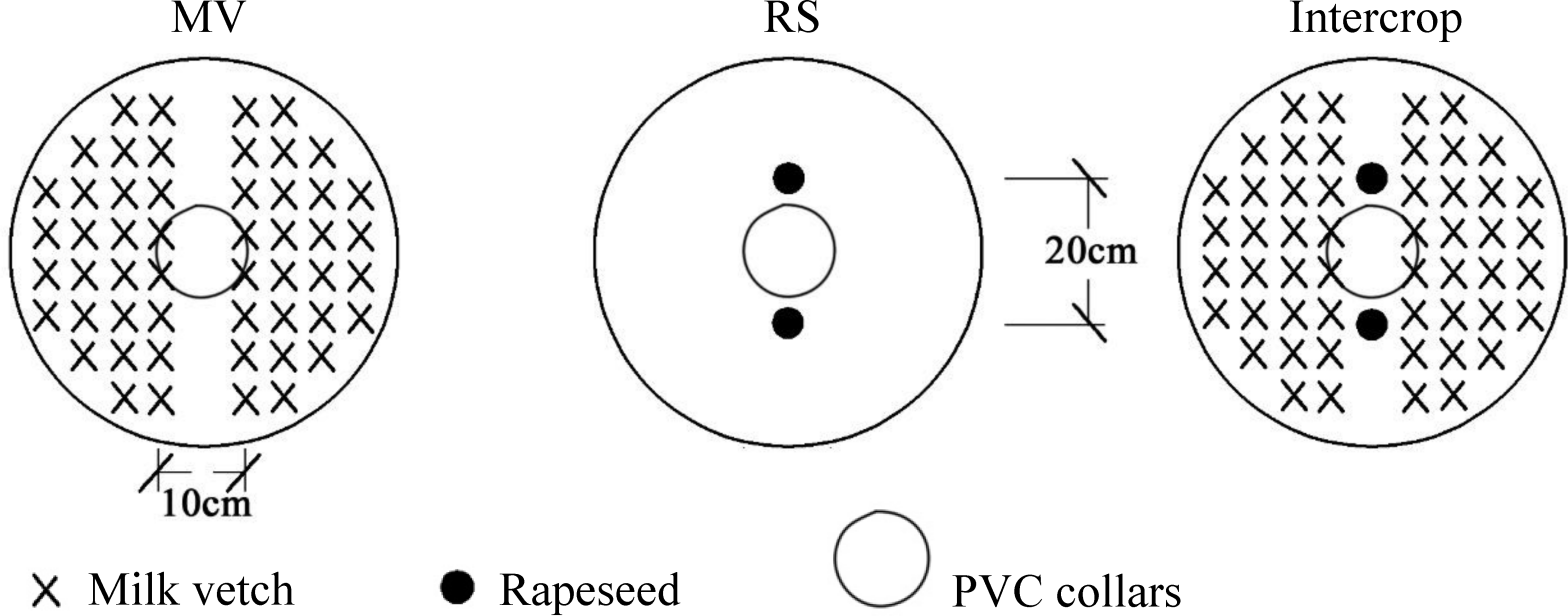
Schematic diagram of the plant cultivation.

Three cropping systems were designed: a) monoculture milk vetch (MV): Leping variety sown by broadcasting with 1.0 g seeds in each bucket; b) monoculture rapeseed (RS): 94005 variety was sown in holes with 2 plants left after seedling emergence; c) milk vetch intercropping with rapeseed (Intercrop): rapeseed was sown in holes with 2 plants after seedling emergence and milk vetch was sown by broadcasting on both sides (0.5 g on each side) (**Figure 2**). Crops were sown in October 2015 and were harvested in May 2016. The experiment had a randomized complete block design with six replicates.

### 2.3 Soil respiration: Soil CO_2_ flux

Soil respiration was quantified using an infrared gas analyzer (Li-Cor 6400xt photosynthesis system installed a 6400-09 soil CO_2_ flux chamber, LI-COR Inc., Lincoln, USA). Cylindrical PVC collars (height, 0.05 m; diameter, 0.11 m) were placed at the core of buckets and inserted one day before measurement to reduce the disturbance of the soil. Each bucket had its own PVC collar. Soil respiration measurements were conducted once per 15 days from 1 November 2015 to 1 May 2016. Each treatment was measured in six replications, and 3 cycles were measured at every turn for each PVC collar. To minimize the influence of the diurnal variation on soil respiration, the measurements were carried out from 9:00 to 11:00 a.m.

### 2.4 Soil hydrothermal factors

Soil temperature (°C) outside the flux chamber at a depth of 5 cm was monitored simultaneously with soil respiration by the infrared gas analyzer. Soil moisture (m^3^ m^-3^) outside the flux chamber at a depth of 5 cm was measured with a handheld multifunction reader (ProCheck connected GS3 sensor, Decagon Inc., USA). The final soil moisture value of each experimental unit was the average of five values taken from the same unit. Each experimental unit's final soil moisture value was the average of five values taken from the same unit.

### 2.5 Statistical analysis

Statistical analysis of all experimental data was conducted using SPSS 17.0, Microsoft EXCEL 2010, and CANOCO 5. Soil respiration data were averaged for each growth stage and were evaluated with two-way ANOVA (two factors of crop system and growth stage). The residuals of the model were checked for normality and homogeneity by Shapiro and Leven’s tests, respectively. If conditions were met, the Tukey test was performed at *P*<0.05. Principal component analysis (PCA) was performed on the soil respiration of crop systems. Detrended correspondence analysis (DCA) and redundancy analysis (RDA) were performed on soil respiration and soil hydrothermal factors. The heterogeneity in soil respiration was tested with a DCA. Due to the gradient length<3.0, a RDA (linear method) was applied.

## 3 Results

### 3.1 Soil CO_2_ efflux

**Figure 3:**
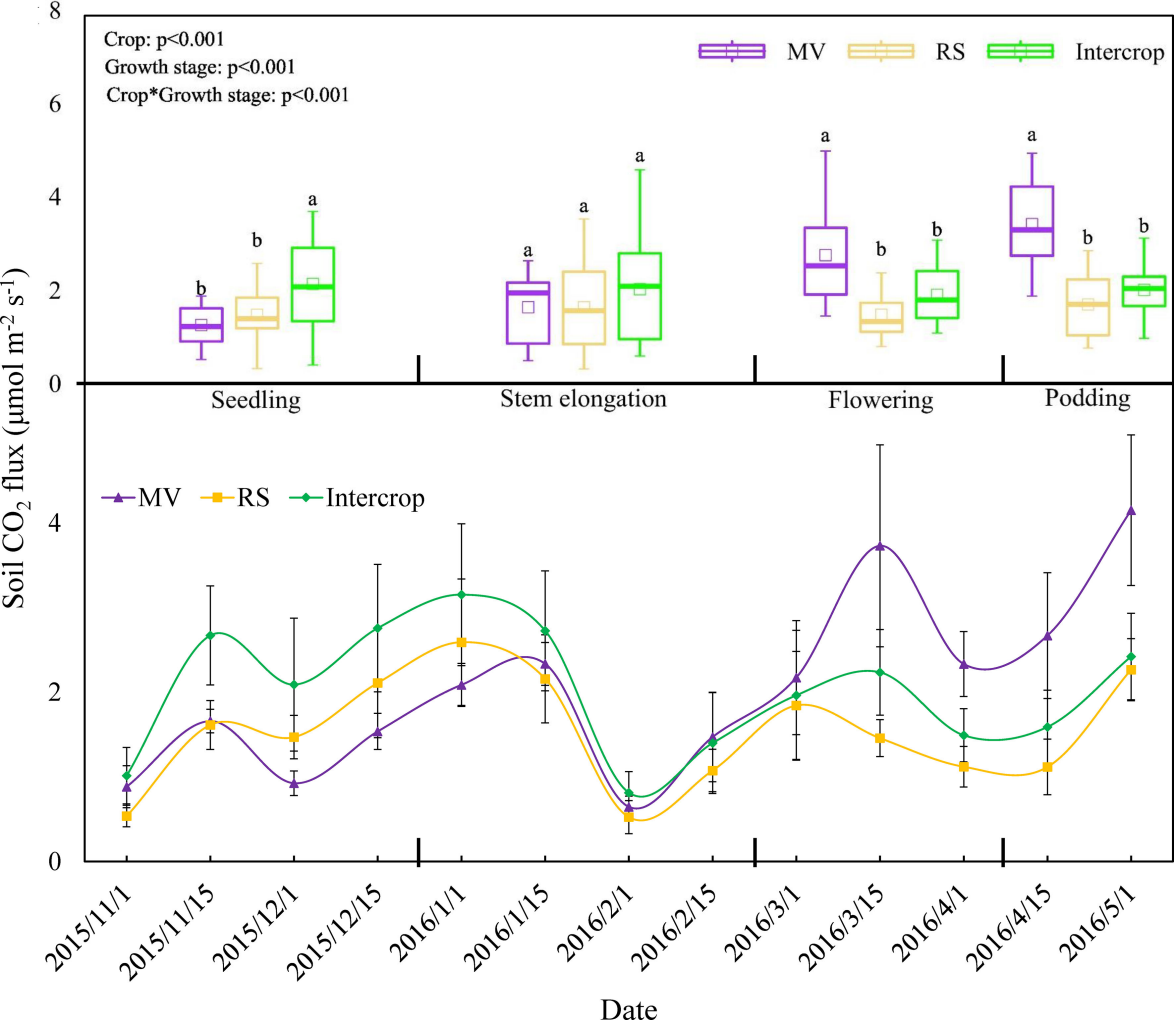
Effects of crop systems on soil respiration. Means are n=24 (seedling and stem elongation stages), n=18 (flowering stage) n=12 (podding stage) (these differences in replicates are because of the length of every growth stage was different) and standard deviations of each growth stage are show. Letters indicate significant differences among crop systems at *P*<0.05.

Soil CO_2_ efflux ranged between 0.53 and 4.15 μmol·m^-2^·s^-1^ during the growing period of crops (**Figure 3**). The turning point of respiration was observed at the lowest temperature during the stem elongation stage, and the pattern of respiration were also depended on the crop system. Soil CO_2_ efflux of MV, RS and Intercrop was 1.44, 1.55, and 2.08 μmol·m^-2^·s^-1^ during seedling and stem elongation stages and 3.08, 1.59, and 1.95 μmol·m^-2^·s^-1^ during flowering and podding stages (**Figure 3**). Soil CO_2_ efflux from Intercrop was 1.4 times higher than from mean of MV and RS at seedling and stem elongation stages, however soil CO_2_ efflux from MV was 1.6 times higher than Intercrop thereafter. The RS and Intercrop had similar CO_2_ efflux rates after the seedling stage, whereas maximum values were found under MV (**Figure 3**).

**Figure 4:**
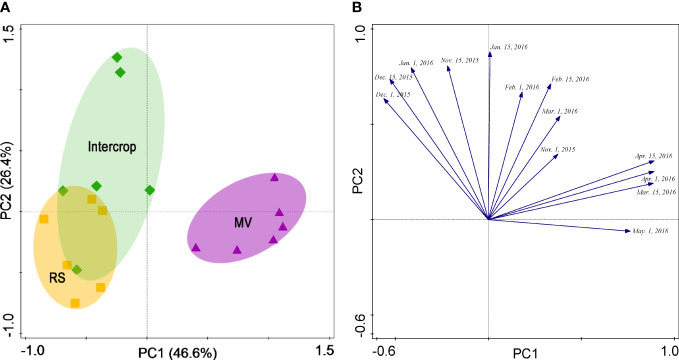
PCA scores **(A)** and corresponding loading values **(B)** for soil respiration (μmol·m^-2^·s^-1^) under various crop systems.

The first two PC explained together more than 80% of the soil respiration variation (**Figure 4A**). The MV was separated from Intercrop along PC1 and PC2 and only along PC1 from RS. In contrast, RS and Intercrop were only weakly separated along the PC2, and no separation along PC1 were found. Soil respiration in three crop systems could be classified into two types (**Figure 4B**). The separation along the PC1 was due to the CO_2_ efflux during the flowering to the podding stage, whereas separation along the PC2 was because CO2 efflux during the seedling and the stem elongation stages. Therefore, the difference in soil respiration between milk vetch and rapeseed was mainly determined by the later period of crop growth.

### 3.2 Soil respiration and hydrothermal factor

**Figure 5:**
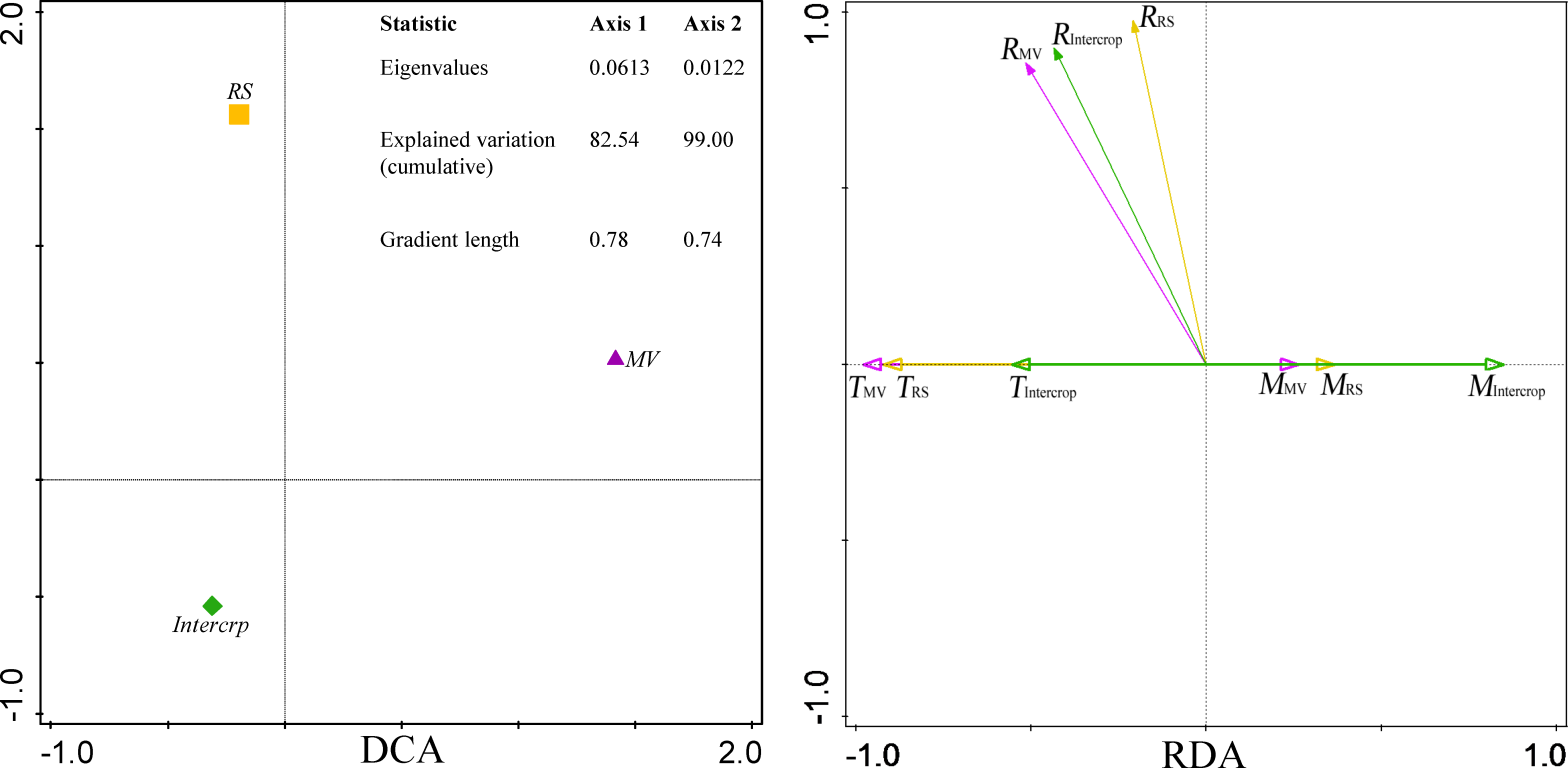
DCA and RDA of soil respiration and hydrothermal factors under various crop systems. DCA was perforemed on soil CO_2_ efflux (μmol m^-2^ s^-1^). RDA was performed on soil CO_2_ efflux (μmol m^-2^ s^-1^), soil temperature (°C), and soil moisture (m^3^ m^-3^). *R*_MV_, *R*_RS_, and *R*_Intercrop_ indicate soil respiration under MV, RS and Intercrop, respectively; *T*_MV_, *T*_RS_, and *T*_Intercrop_ indicate soil temperature; *M*_MV_, *M*_RS_ and *M*_Intercrop_ indicate soil moisture.

According to the DCA, soil respiration in MV, RS and Intercrop was found to be heterogeneous (gradient length<3.0). RDA showed that soil respiration was positively correlated with soil temperature and negatively with moisture (**Figure 5**). However, differences between crop systems were still observed. Firstly, MV had the closest correlation between soil respiration and hydrothermal factors, followed by Intercrop and RS. Secondly, the correlation between soil respiration and the temperature was more substantial than with soil moisture in the case of MV and RS; in the case of Intercrop, the correlation between soil respiration and moisture was stronger than that with temperature (**Figure 5**). This indicated that Intercrop changed the responses of soil respiration to hydrothermal factors.

**Figure 6:**
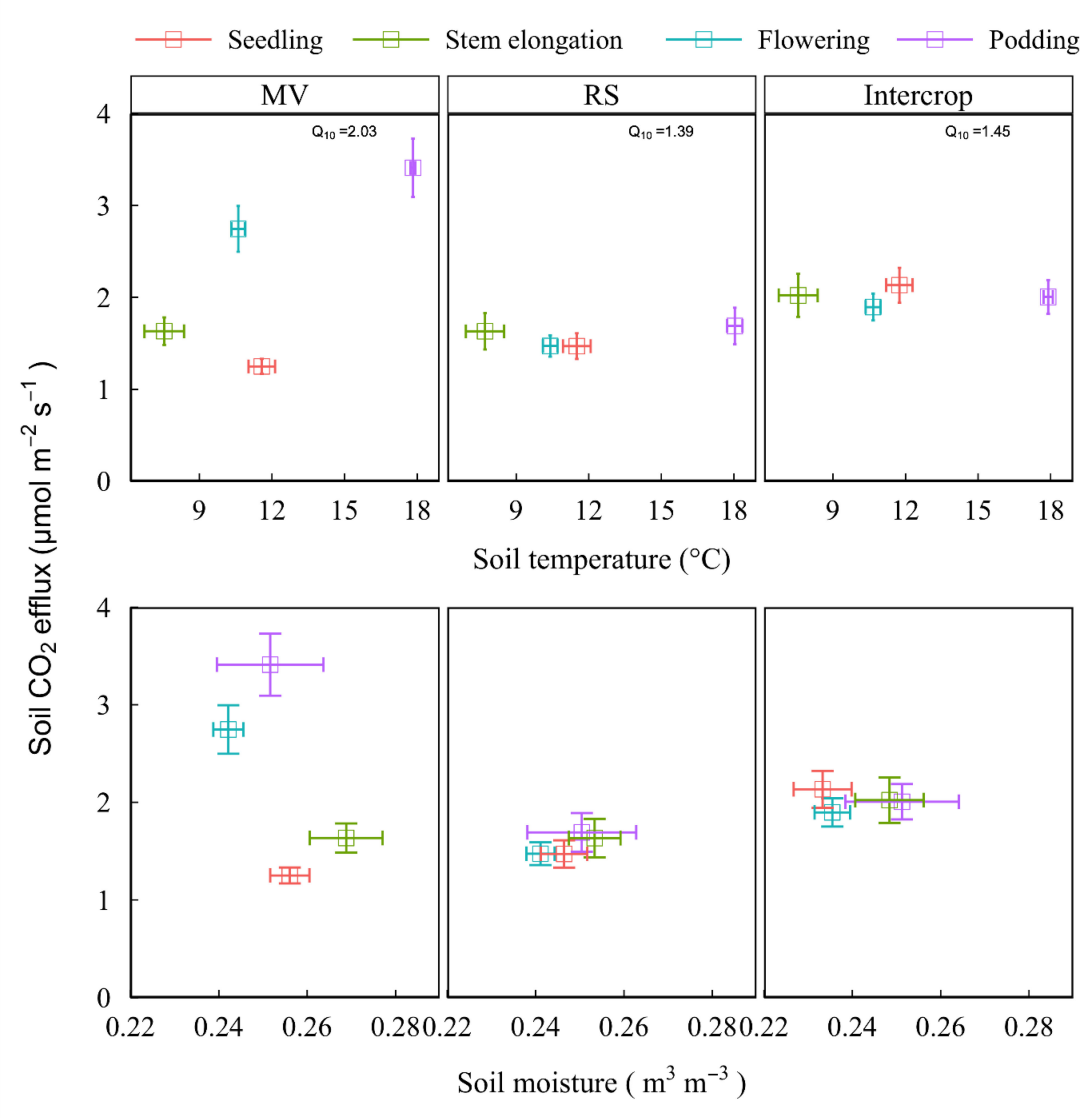
Soil respiration and hydrothermal factor under various crop systems and plant growth stages. Data shows means and standard errors. Q_10_ value is the sensitivity of soil respiration to temperature change, which was calculated as y=ae^bx^, Q_10_ = e^10b^, where y is the soil respirartion, and x is temperature, a and b are fiited parameters.

The Q_10_ values were 2.03, 1.39, and 1.45 in MV, RS, and Intercrop, respectively. Sensitivity of soil respiration to the temperature was lower in Intercrop than in MV and was independent on temperature changes, namely, although there was the same high temperature at the flowering and podding stages, the CO_2_ emission rates from Intercrop and RS were lower than from MV (**Figure 6**). Intercrop also decreased soil moisture, especially at the seedling and stem elongation stages, compared to the monoculture; soil CO_2_ efflux was smaller in RS and Intercrop than in MV when soil moisture was low. The CO_2_ efflux from RS and Intercrop was more constant under the moisture fluctuations compared with MV (**Figure 6**), which indicates that both RS and Intercrop reduced the response of soil respiration to moisture.

## 4 Discussion

The high CO_2_ efflux from Intercrop at the early growth stages are explained by low competition for the soil resources between species (Mushagalusa et al., 2008), and intensive plant growth. Intercropping with RS may decrease the impact of MV on soil respiration at the later stage (**Figure 3**) i) due to inhibition of MV growth (Zhou et al., 2018), thus, decreasing the release of root exudates from MV into the soil (Liu et al., 2013) and ii) due to suppress microbial biomass and activity in the RS rhizosphere in the presence of MV (Zhou et al., 2019b), and thus, reducing rhizosphere respiration (Blaise et al., 2021; Yang et al., 2021). The RS and Intercrop had similar CO_2_ efflux rates after the seedling stage, whereas maximum values were found from MV (**Figure 3**). This is explained by higher root exudation under N_2_ fixing plant species (Zúñiga-Feest et al., 2018) and thus, higher rhizosphere respiration (Becker and Holz, 2021). This showed the positive effect of intercropping on the SOM accumulation because the CO_2_ efflux was reduced compared to N-fixing plant monoculture.

Seasonal and interannual variations of CO_2_ emission are related to the soil temperature and moisture (Suseela et al., 2012) because these parameters directly regulate microbial biomass and activity (Zhou et al., 2019b). Sensitivity of soil respiration to temperature was lower in Intercrop than in MV and was independent of temperature changes (**Figure 6**). Intercrop also decreased soil moisture, especially at the seedling and stem elongation stages, compared to the monoculture (**Figure 6**). This explains why in the Intercrop the growth of MV was suppressed (Zhou et al., 2018). High CO_2_ emission rate under MV observed at the flowering and podding stages can be explained more by the high soil moisture at the initial plant growth stages, then by the temperature. In contrast, the decreased CO_2_ efflux rate under Intercrop at later stages can be directly affected by variations in moisture or temperature, and probably other factors, such as changes in the composition of microbial communities (Zhou et al., 2019b), a decrease of rhizosphere C flux (Suseela and Dukes, 2013) and plant species-species interactions (Dijkstra et al., 2010).

The similar trends in CO_2_ efflux and temperature suggested that soil temperature was still the most important factor affecting soil respiration (**Supplementary Figure 1**). Soil respiration was positively correlated with soil temperature, and a negative correlation of soil respiration to soil moisture was also found, especially in Intercrop (**Figure 5**). It illustrated that the effect of soil moisture on soil respiration is more important in intercropping systems than in monoculture. Soil respiration responses to increases in temperature are constrained by soil moisture (Conant et al., 2004). If the soil moisture is often lower than the soil water holding capacity, the soil respiration cannot be high enough to reach the limiting point due to reduced oxygen diffusion into the soil and inhibited substrate decomposition (Tang et al., 2006; Hu et al., 2017). Furthermore, as microbial respiration is linearly related to soil water content and log-linearly related to water potential (Cook and Orchard, 2008), the decreased soil moisture will directly lead to the decrease of soil microbial biomass and functional activity (Zhou et al., 2019b). Thus, the variation in soil moisture can be the real reason for inhibited soil respiration by milk vetch intercropping with rapeseed.

## 5 Conclusions

Soil CO_2_ efflux from Intercrop was 1.4 times higher than from mean of MV and RS at seedling and stem elongation stages, however, soil CO_2_ efflux from MV was 1.6 times higher than Intercrop after that. Cultivation of legume in monoculture, although there is a positive contribution to soil N balance, can promote SOM losses compared to Brassica. In contrast, intercropping of Legume with Brassica is a beneficial agricultural practice to reduce the rate of CO_2_ efflux, which is related to the flowering and podding stages of plant growth. The sensitivity of soil respiration to temperature decreased in Intercrop, in which the variation of soil moisture was the primary factor to inhibit soil respiration. Therefore, milk vetch-rapeseed intercropping could be a potential approach to produce low CO_2_ emissions from farmland, however soil moisture should be adequately regulated so that agricultural intercropping systems can be well adaptable in the face of frequent global droughts.

## Data availability statement

The original contributions presented in the study are included in the article/**Supplementary Material**. Further inquiries can be directed to the corresponding author.

## Author contributions

All authors contributed to the study conception and design. Material preparation, data collection and analysis were performed by QZ, AG and JC. The first draft of the manuscript was written by QZ and all authors commented on previous versions of the manuscript. All authors contributed to the article and approved the submitted version.
