# Supplementary figures and images for: Reduction in soil CO2 efflux through alteration of hydrothermal factor in milk vetch (Astragalus sinicus L.)-rapeseed (Brassica napus L.) intercropping system
Source: Front Plant Sci. 2023 Jan 10;13:1093507. doi: 10.3389/fpls.2022.1093507 (PMC9875911; doi:10.3389/fpls.2022.1093507)

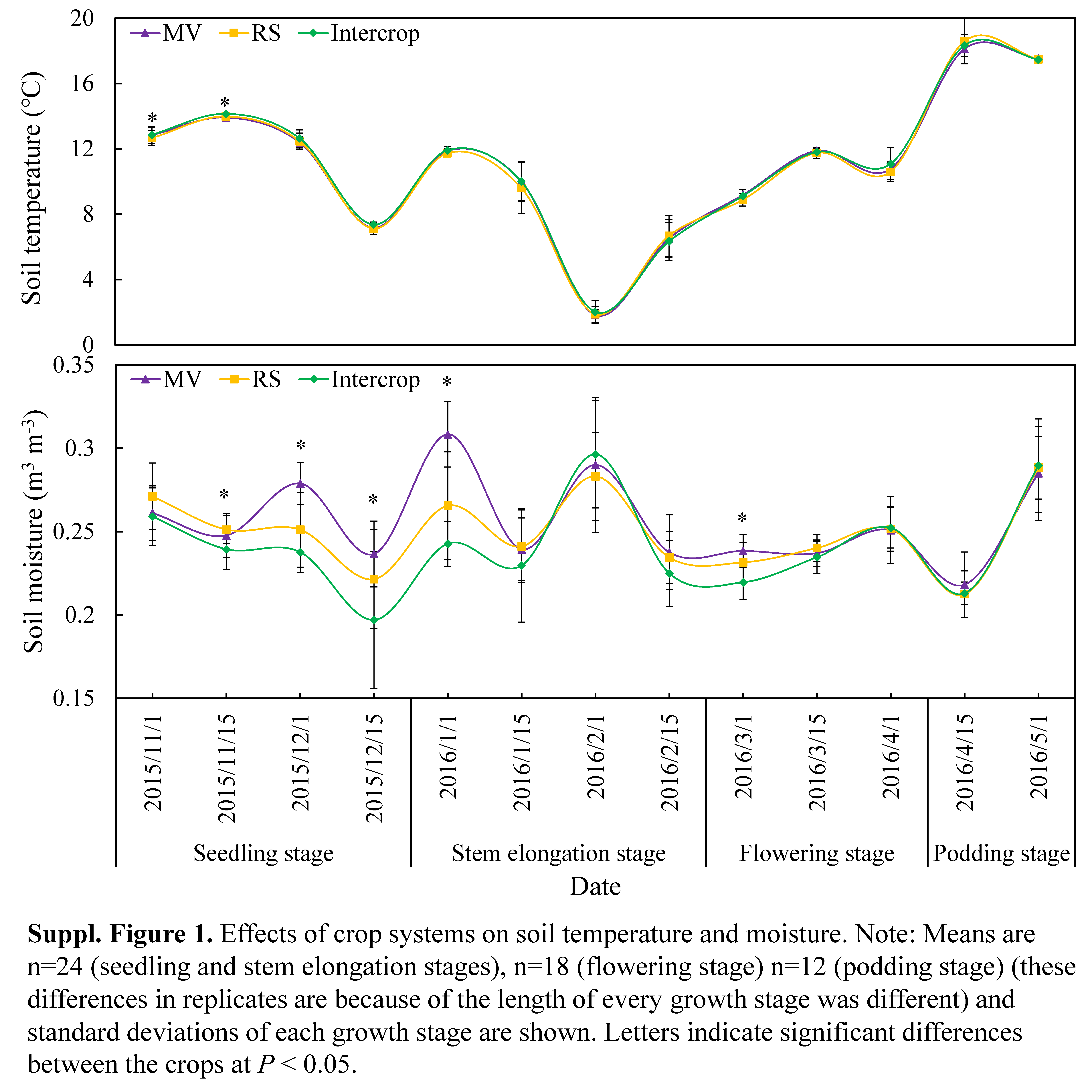

Supplement: Supplementary file 1 [file Image_1.tiff]
